# Waiting time at health facilities and social class: Evidence from the Indian caste system

**DOI:** 10.1371/journal.pone.0205641

**Published:** 2018-10-15

**Authors:** Mujaheed Shaikh, Marisa Miraldo, Anna-Theresa Renner

**Affiliations:** 1 Health Economics and Policy, Vienna University of Economics and Business, Vienna, Austria; 2 Department of Management, Imperial College Business School, Imperial College London, London, United Kingdom; 3 Centre for Health Economics and Policy Innovation, Imperial College Business School, Imperial College London, London, United Kingdom; Medical University Graz, AUSTRIA

## Abstract

Waiting time for non-emergency medical care in developing countries is rarely of immediate concern to policy makers that prioritize provision of basic health services. However, waiting time as a measure of health system responsiveness is important because longer waiting times worsen health outcomes and affect utilization of services. Studies that assess socio-economic inequalities in waiting time provide evidence from developed countries such as England and the United States; evidence from developing countries is lacking. In this paper, we assess the relationship between social class i.e. caste of an individual and waiting time at health facilities—a client orientation dimension of responsiveness. We use household level data from two rounds of the Indian Human Development Survey with a sample size of 27,251 households in each wave (2005 and 2012) and find that lower social class is associated with higher waiting time. This relationship is significant for individuals that visited a male provider but not so for those that visited a female provider. Further, caste is positively related to higher waiting time only if visiting a private facility; for individuals visiting a government facility the relationship between waiting time and caste is not significant. In general, caste related inequality in waiting time has worsened over time. The results are robust to different specifications and the inclusion of several confounders.

## Introduction

Waiting time for non-emergency medical care as a measure of responsiveness is an important policy issue in developed countries and health care systems with universal coverage [1,2]. In contrast, in developing and low-income countries, it is rarely of immediate concern to policy makers. This is perhaps not surprising given that health systems in such countries often focus on prioritizing provision of basic preventive and curative services, and reducing catastrophic expenditures. Furthermore, low utilization rates and the general consensus that waiting time is contingent on individuals accessing and utilizing health services, leads policy makers to prioritize access and utilization. However, waiting time is equally important in developing countries because longer waiting times might worsen health outcomes, reduce treatment benefits, and negatively affect desire to visit health facilities subsequently [3].

High waiting times might exist due to limited supply side factors such as low density of medical staff and sparsely located health facilities, or as demand rationing mechanism [4]. In either case the distribution of waiting times across different sub-groups of the population might generate inequalities of access, utilization and health outcomes. If rationing is based on factors other than clinical need such as ability to pay, education or race, it has detrimental equity implications. Socio-economically disadvantaged groups often have poor health. If waiting time not only directly affects health through delayed utilization, but also reduces future utilization, the foregone care, further aggravates the divide in health status between the socio-economic advantaged and disadvantaged groups. Furthermore, the impact of the opportunity cost of waiting longer (foregone earnings for example) may be much higher for socio-economically disadvantaged individuals.

In this paper, we assess waiting time at health facilities (for outpatient services) in India and how it relates to an important determinant of socio-economic class in India, the caste of an individual. The caste system is a 3000 year old hierarchical system which divides the Indian population into different social classes (initially four that later extended to five). The spot at the top of the hierarchy is taken by the ‘Brahmins’ and the lowest category are the so-called ‘Ati-Sudras’ or the ‘Untouchables’. The official caste divisions that exist now are slightly different and the available data simplify the task of dividing them into Brahmins, Forward caste, Other backward castes (OBC), Scheduled castes (SC), Scheduled tribes (ST), and Other castes. The data section describes each of these categories in more detail.

We focus on caste-based differences in waiting time in this paper for several reasons. First, caste is seen as a strong determinant of social class, occupation, exclusion and exploitation in India, even more so than income [5]. Caste is crucial in determining individual outcomes in almost all spheres of life [6]. It affects several aspects of an individual’s life–restrains upward mobility, reduces education opportunities, affects work and income, dictates residence and living conditions, and in some parts of the country even restricts access to the most basic rights such as access to clean drinking water. Second, the government of India initiated several affirmative actions to specifically reduce caste-based inequalities and provide everyone with equal opportunity–one can argue that this has led to positive discrimination [7]. However, even today, 68 years after independence, caste-based discrimination can be observed in both rural and urban parts of the country. A few studies analyze the relationship between caste and health outcomes and report higher mortality rates and lower life expectancy, lower subjective health status, greater financial burden of health care, lower immunization rates, and lower utilization among lower castes. Some have found inequalities in access to health services and health services utilization measures such as immunization and use of preventive and maternal service [6,8–12]. Third, evidence suggests that even in the most egalitarian societies in India significant inter-caste disparities still exist [7]. And finally, while it may seem that caste is a phenomenon unique to India, this paper is relevant to other developing countries which have multiple minority groups—e.g. China with groups other than the Han Chinese population, different tribal groups within the African regions, different religious minorities within a religion such as Sunnis and Shias within Islam.

While there are several studies that assess the extent and determinants of inequality in utilization and health outcomes (e.g. [13–22]) there are relatively fewer studies that assess this in terms of a responsiveness measure such as waiting time (e.g. [23–27]). Most studies that assess socio-economic inequalities in waiting time provide evidence from developed countries such as England, Australia and the United States but do not focus on developing countries where waiting times might impact the population more severely [2]. Further, while fixed characteristics such as race, ethnicity and gender of patients have received substantial attention in the utilization, health and social outcomes literatures (e.g. [14,15,17,28]) they have received relatively little attention in the assessment of the determinants of waiting times and other aspects of health systems responsiveness.

We add to the above literature by focusing on an important client orientation dimension of health system responsiveness. Assessment of racial/ethnic disparities in terms of non-clinical measures has largely focused on the US context (e.g. [29–31]). However, such non-need characteristics as determinants of responsiveness may well extend to both developed and developing countries where not only minority ethnic groups may face discrimination, but also different minority groups within the same ethnicity may face discrimination.

To this end, we use household level data with a sample size of 27,251 households in each round (2005 and 2011) of the Indian Human Development Survey (henceforth IHDS) and assess how caste relates with waiting time in the two rounds. We compare the relationship between waiting time and caste between the two rounds and assess heterogeneity in this relationship by provider gender and by provider type i.e. government versus private facilities. This is the first paper to the best of our knowledge that presents evidence on social class based differences in waiting time from a developing country. The results are informative especially to those countries, which have multiple minority groups and provide further motivation to conduct such studies within these countries and address such grievances if true universal coverage is the larger aim.

The paper proceeds as follows. In sections 2 and 3 we present the data and empirical methods, respectively. Section 4 presents the results and section 5 the concluding remarks.

## Methods

### Data

We use household data from two waves of the IHDS [32, 33]; the first wave was conducted in 2004–05 and the second wave that re-interviewed most of the previous households was conducted in 2011–12. The interviews are conducted at the household level typically with the head of the household.

The IHDS is a nationally representative survey. The collected data and detailed information is publicly available from: https://ihds.umd.edu/. 41,554 households were interviewed in the first round, in both urban and rural areas across several states, districts, and villages in India. Almost 83% of these households were re-interviewed in the second round. We include only those households in the analysis that are observed in both rounds since this allows us to assess changes in caste-based differences in waiting time from 2005 to 2012 within the same entity. Households that were new additions in the second round and households that split from the focal household were excluded from the analysis. Further, observations with missing values on one or more of the relevant variables for the analysis were excluded leading to a final sample size of 27,251 households in each wave.

The survey contains different modules and was translated into 13 Indian languages. The sample was drawn using stratified random sampling. The large sample size lends us the advantage of analyzing different segments of the population besides other advantages of power and precision [32]. The IHDS is the only national survey in India that provides detailed information on income, education, health and well-being and most importantly, information on health facilities [32]. Therefore, it provides us with a unique opportunity to assess caste-based disparities in health-related variables.

### Variable description

We describe the relevant variables next; Table 1 shows descriptive statistics for all the variables included in the analysis for both rounds, respectively.

**Table 1:** Summary statistics.

	**Round 1**								
	*Total*	*Brahmin*		*Forward caste*	*OBC*	*SC*	*ST*	*Other*	
Observations	27,251	1,474		4,571.00	10,910	5,896	2,082	2,318	
Percentage of total	100	5.4		16.8	40.0	21.6	7.6	8.5	
**Waiting time**									
Waiting time (minutes)	21.82	18.53		22.16	21.78	23.14	19.18	22.47	
Any waiting time (share)	0.90	0.84		0.89	0.91	0.90	0.85	0.92	
Conditional waiting time (minutes)	24.25	22.05		24.82	23.85	25.62	22.45	24.31	
**Sociodemographic factors**									
No schooling (share)	0.21	0.04		0.13	0.20	0.30	0.38	0.17	
Primary school (share)	0.15	0.06		0.11	0.16	0.18	0.19	0.14	
Secondary school (share)	0.38	0.36		0.38	0.40	0.35	0.31	0.41	
Above secondary school (share)	0.26	0.55		0.39	0.23	0.17	0.12	0.29	
Household income (median Rs.)	51428.37	58,424.35		47,300	28,880.46	24,775.82	21,204.5	42,000	
Rural (share)	0.69	0.53		0.63	0.70	0.75	0.88	0.56	
**Type of minor illness**									
Fever (share)	0.55	0.51		0.50	0.56	0.57	0.60	0.50	
Cough/cold (share)	0.11	0.12		0.14	0.10	0.11	0.10	0.11	
Diarrhoea (share)	0.05	0.04		0.05	0.05	0.05	0.07	0.05	
Other (share)	0.29	0.33		0.31	0.30	0.27	0.23	0.34	
**Religion**									
Hindu (share)	0.81	1.00		0.63	0.85	0.91	0.79	0.59	
Muslim (share)	0.12	0.00		0.23	0.12	0.01	0.00	0.30	
Christian (share)	0.03	0.00		0.05	0.01	0.02	0.08	0.06	
Sikh (share)	0.03	0.00		0.07	0.01	0.04	0.00	0.04	
Buddhist (share)	0.01	0.00		0.00	0.00	0.03	0.01	0.00	
Jain (share)	0.00	0.00		0.01	0.00	0.00	0.00	0.01	
Tribal (share)	0.01	0.00		0.00	0.00	0.00	0.11	0.00	
Others (share)	0.00	0.00		0.00	0.00	0.00	0.00	0.00	
None (share)	0.00	0.00		0.00	0.00	0.00	0.00	0.00	
**Health care provider**									
Female (share)	0.149	0.13		0.14	0.15	0.15	0.15	0.16	
Government provider (share)	0.295	0.27		0.26	0.28	0.32	0.41	0.30	
	**Round 2**								
	*Total*		*Brahmin*	*Forward caste*	*OBC*	*SC*	*ST*		*Other*
Observations	27,251		1,452	6,158	11,063	5,993	2,159		426
Percentage of total	100		5.3	22,6	40.6	22	7.9		1.5
**Waiting time**									
Waiting time (minutes)	28.62		25.30	29.41	28.23	30.33	24.71		34.41
Any waiting time (share)	0.94		0.91	0.95	0.94	0.95	0.93		0.99
Conditional waiting time (minutes)	30.34		27.79	30.90	29.98	32.09	26.45		34.90
**Sociodemographic factors**									
No schooling (share)	0.17		0.04	0.10	0.17	0.24	0.30		0.14
Primary school (share)	0.14		0.06	0.10	0.14	0.16	0.18		0.13
Secondary school (share)	0.36		0.31	0.34	0.38	0.36	0.32		0.38
Above secondary school (share)	0.33		0.59	0.46	0.31	0.24	0.20		0.35
Household income (median Rs.)	77,300		118,897.5	103,470	73,630	68,600	51,000		90,650
Rural (share)	0.66		0.54	0.59	0.66	0.71	0.86		0.59
**Type of minor illness**									
Fever (share)	0.65		0.67	0.61	0.66	0.68	0.62		0.62
Cough/cold (share)	0.09		0.08	0.11	0.08	0.09	0.11		0.11
Diarrhoea (share)	0.03		0.03	0.03	0.03	0.03	0.03		0.03
Other (share)	0.23		0.22	0.25	0.23	0.21	0.24		0.25
**Religion**									
Hindu (share)	0.82		0.99	0.68	0.82	0.92	0.81		0.54
Muslim (share)	0.12		0.00	0.21	0.15	0.01	0.01		0.36
Christian (share)	0.03		0.00	0.04	0.02	0.02	0.10		0.08
Sikh (share)	0.03		0.00	0.06	0.01	0.04	0.00		0.00
Buddhist (share)	0.01		0.00	0.00	0.00	0.02	0.01		0.01
Jain (share)	0.00		0.00	0.01	0.00	0.00	0.00		0.01
Tribal (share)	0.00		0.00	0.00	0.00	0.00	0.06		0.00
Others (share)	0.00		0.00	0.00	0.00	0.00	0.01		0.00
None (share)	0.00		0.00	0.00	0.00	0.00	0.00		0.00
**Health care provider**									
Female (share)	0.16		0.12	0.16	0.17	0.15	0.14		0.37
Government provider (share)	0.31		0.28	0.28	0.30	0.34	0.41		0.42

### Caste

As mentioned early on, the Indian populace can be divided into different social classes based on caste. Caste is primarily a Hindu phenomenon; however, it may be relevant for a very small proportion of individuals from other religions that, among other reasons, may either be converts or have occupations that were specific to a caste and might wish to retain eligibility for affirmative schemes. We control for religion in our analysis and in robustness tests also exclude religions other than Hindu. Officially, and in the survey the caste variable provides us with six categories. Brahmins are considered the uppermost class followed by the Forward class that is not eligible for any affirmative schemes but are not classified as Brahmins either. The next three classes (OBC, SC and ST) are somewhat less distant in terms of social hierarchy but nevertheless are eligible for government schemes and reservations. For example, scheduled castes were former untouchable castes while the scheduled tribes are economically and socially marginalized [7]. The final group is all others that do not report any of the former caste groups. We keep Brahmins as the reference category throughout the analysis and compare outcomes with respect to this group.

It is important to note that in the first round of the survey, the ‘Forward caste’ category did not exist, thus there were only five caste categories. In the second round however, ‘Forward caste’ was included as an additional category. We assume that individuals that chose ‘Forward’ in the latter round, reported ‘Other’ in the first round due to lack of the ‘Forward caste’ option in the first round. For data comparability between the two rounds, we therefore include the additional category ‘Forward caste’ in the first round for such respondents. OBCs and SCs consist of 40 and 22 percent of the total sample in both rounds, respectively. The Forward caste is the next largest group (17 percent and 23 percent in round one and two, respectively) followed by Scheduled tribes (8 percent in both rounds). Brahmins compose about 5 percent of the sample in both waves. Note that the descriptive statistics show that there is no variation across rounds in the proportion of people belonging to a particular caste for almost all categories. However, for some we do observe slight differences due to people reporting different caste categories in round 1 and 2. While it is plausible to presume that individuals could theoretically change caste, we perform a sensitivity analysis by excluding such observations from the regressions. The results are robust to this. These proportions are similar to the ones reported by others and representative of the Indian population [6,7].

### Waiting time

**Fig 1:**
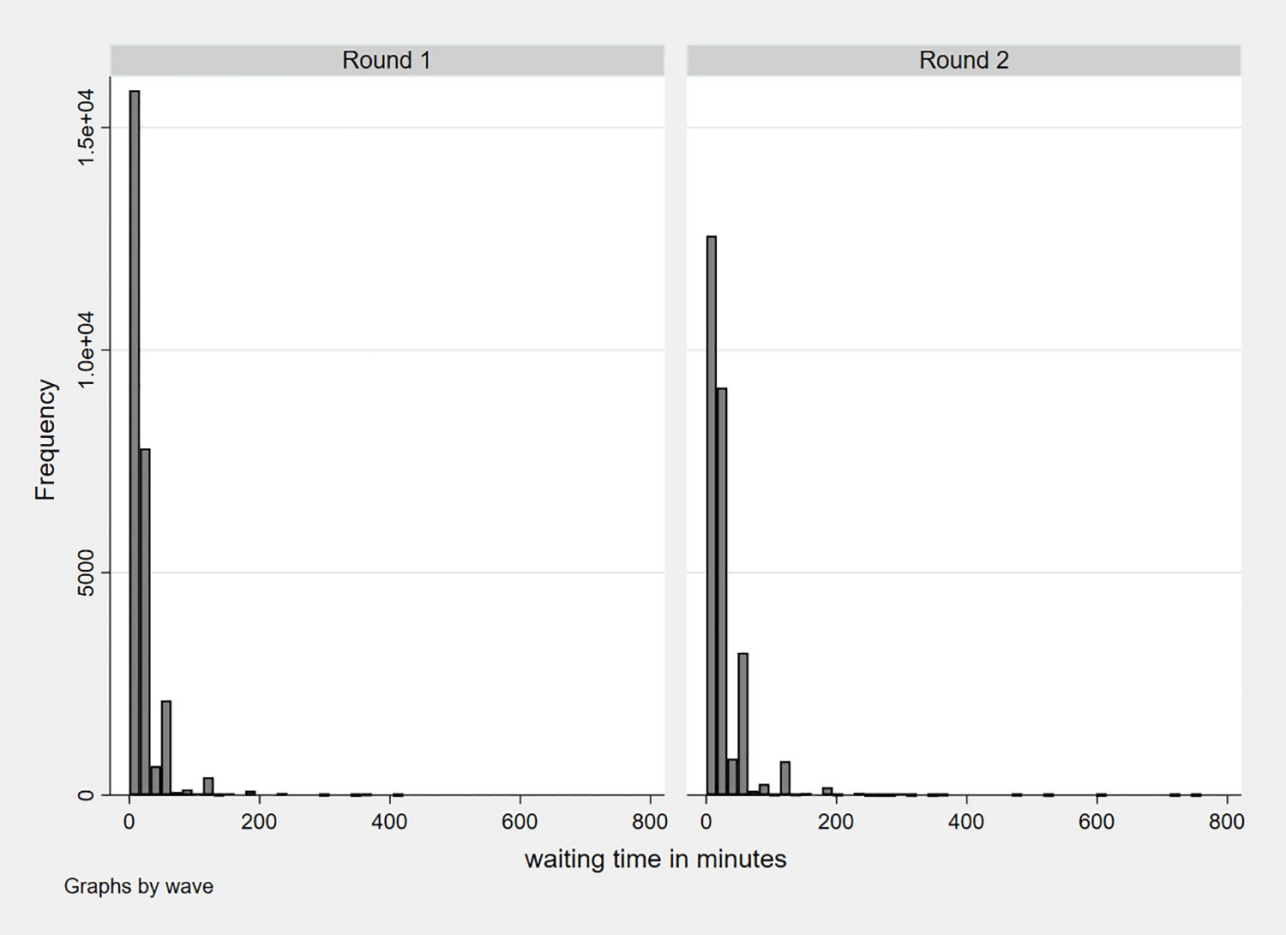
Waiting time distribution in both rounds.

Waiting time is reported in minutes for outpatient services only. In the survey respondents are asked different questions related to quality of care at the health facility in their last visit, for a minor illness for themselves or their children. Within these questions, respondents are asked about waiting time that is measured by how long they usually have to wait at the facility they visited. The descriptive statistics in Table 1 show that the average waiting time increased from 22 minutes in round one to 29 minutes in round two (t-statistic = -25.24, p < 0.0001); 10 percent report no waiting time (i.e. 0 minutes) in round one which decreased to 6 percent in round two. Fig 1 shows the distribution of waiting time in both rounds. It becomes evident that the distribution is highly left-skewed with a majority of respondents reporting a waiting time below 200 minutes (99.7 percent in round 1 and 99.5 percent in round 2).

**Fig 2:**
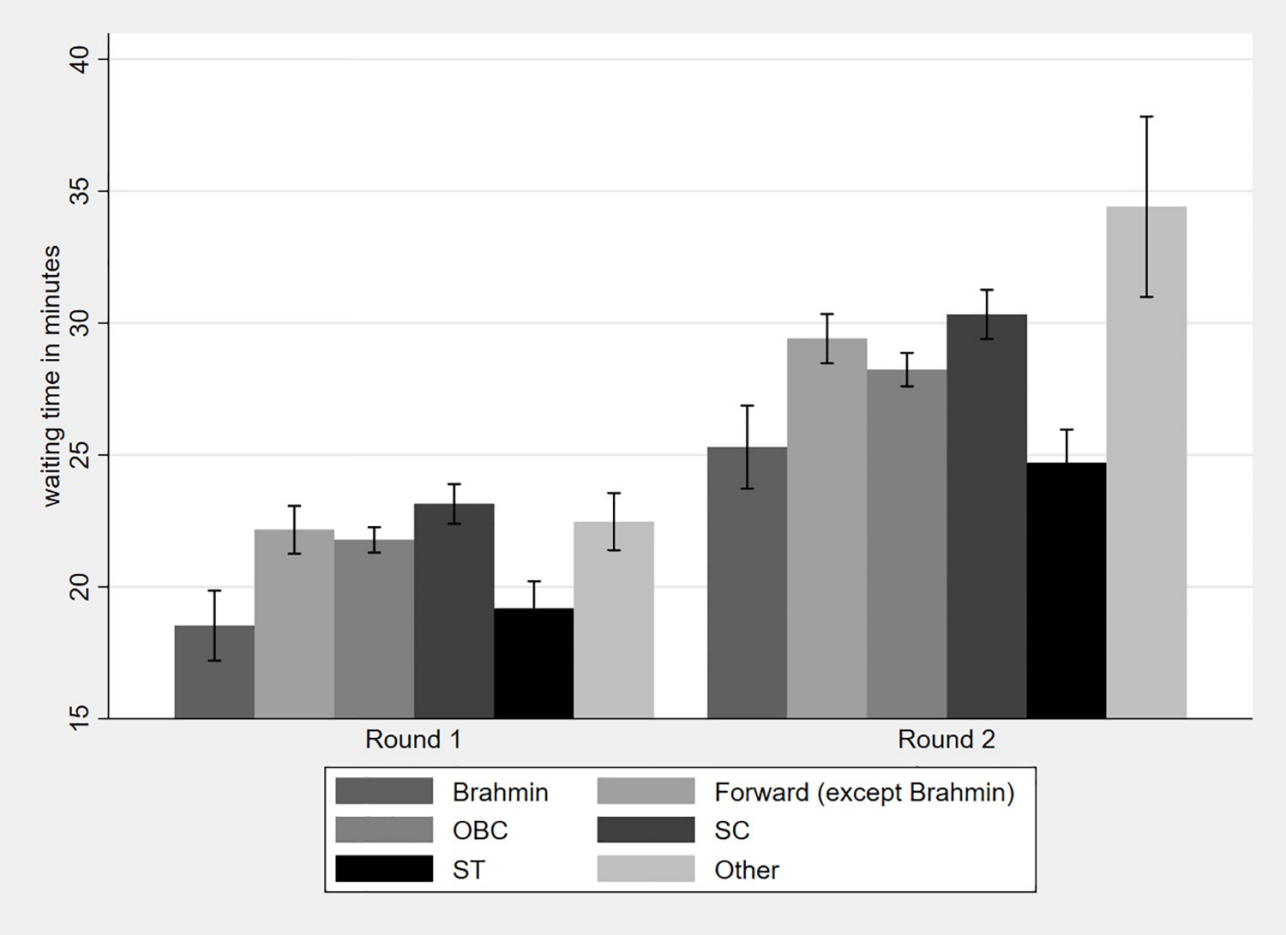
Average waiting time by caste categories.

Average waiting time for all caste categories increased from round one to round two with a statistically significant difference in the waiting time for each caste between the two waves (see Table A in S1 Appendix). Fig 2 shows waiting time by caste categories for both rounds with the corresponding confidence intervals (95% CI). Within each wave, Brahmins on average have lower waiting times compared to the other castes. The difference in waiting time is statistically significant between Brahmins and all other castes in both rounds, with the exception of STs. Overall, the differences in the average waiting times between other castes are not as stark and consistently significant as the difference between Brahmins and all other castes. Table B in S1 Appendix shows the comparison between the waiting times of all castes using Bonferroni adjusted multiple comparison testing. Similarly, the percentage of respondents reporting no waiting time at all is the highest for Brahmins in both rounds (16 and 9 percent, respectively).

### Control variables

Social class has been found to be positively associated with income and education which are also important determinants of how physicians and health workers respond to patients [5,7,34,35]. We therefore control for household income and education level. The data for income comes from the ‘HH INCOME’ variable provided within the IHDS that represents the household annual income in Rupees. Despite careful collection of over 50 sources of income, measurement error cannot be ruled out (see Desai et al., 2010 for a detailed description of the sources of error in measuring income) [5]. The education variable is proxied by the highest education level achieved by an adult member within the household. We categorize this into no education, primary, secondary, and higher secondary education. As mentioned earlier (footnote 2), although the caste system is mainly relevant to Hindus, we control for religion throughout the analysis. In addition, we include a control for place of residence (rural vs. urban) to account for differences in health care provision and supply-side constraints across more and less densely populated areas.

Finally, health need may also directly determine waiting time in that sicker individuals are prioritized compared to others. Concurrently, worse health may be associated with lower castes and may also be associated with the treatment meted out to individuals of lower castes [6,8–12,36,37]. Therefore, severity of illness may lead to a downward bias in the estimates. In the IHDS, respondents report the type of minor illness for which they visited the health facility–i.e. fever, cough, cold, diarrhea and others. We use this information to construct dummy variables for illness type and include them in all regressions as controls.

### Heterogeneity

We also assess heterogeneity in two ways. Prior research argues that inherent bias or prejudice may exist from the provider’s side giving rise to health inequalities. As such, physicians operate with prior beliefs, and characteristics such as gender, race or ethnicity form an important part of how providers feel for patients and even their medical decisions [38]. Patients also form expectations with regards to physician gender [39]. The literature on physician gender and patient care provides evidence that female physicians show lower implicit bias, engage in more patient centered approaches and communication, and lead to more participatory patient visits [31,40–42]. We therefore assess differences in waiting time by gender of the provider at the health facility. While most respondents see a male health care worker, about 15 and 16 percent see a female health care worker in the two rounds, respectively. We excluded a small fraction of individuals that see both a male and female provider.

We also consider the type of provider consulted, i.e. public or private health facility. The type of provider is a dummy variable where one corresponds to seeing a Public provider defined as ‘a government doctor or nurse’, and zero corresponds to seeing a private provider i.e. ‘a government doctor or nurse in private, private doctor or nurse, chemists or other healers.’ The fraction of respondents visiting a government facility is almost the same in both the rounds (approx. 30 percent).

**Fig 3:**
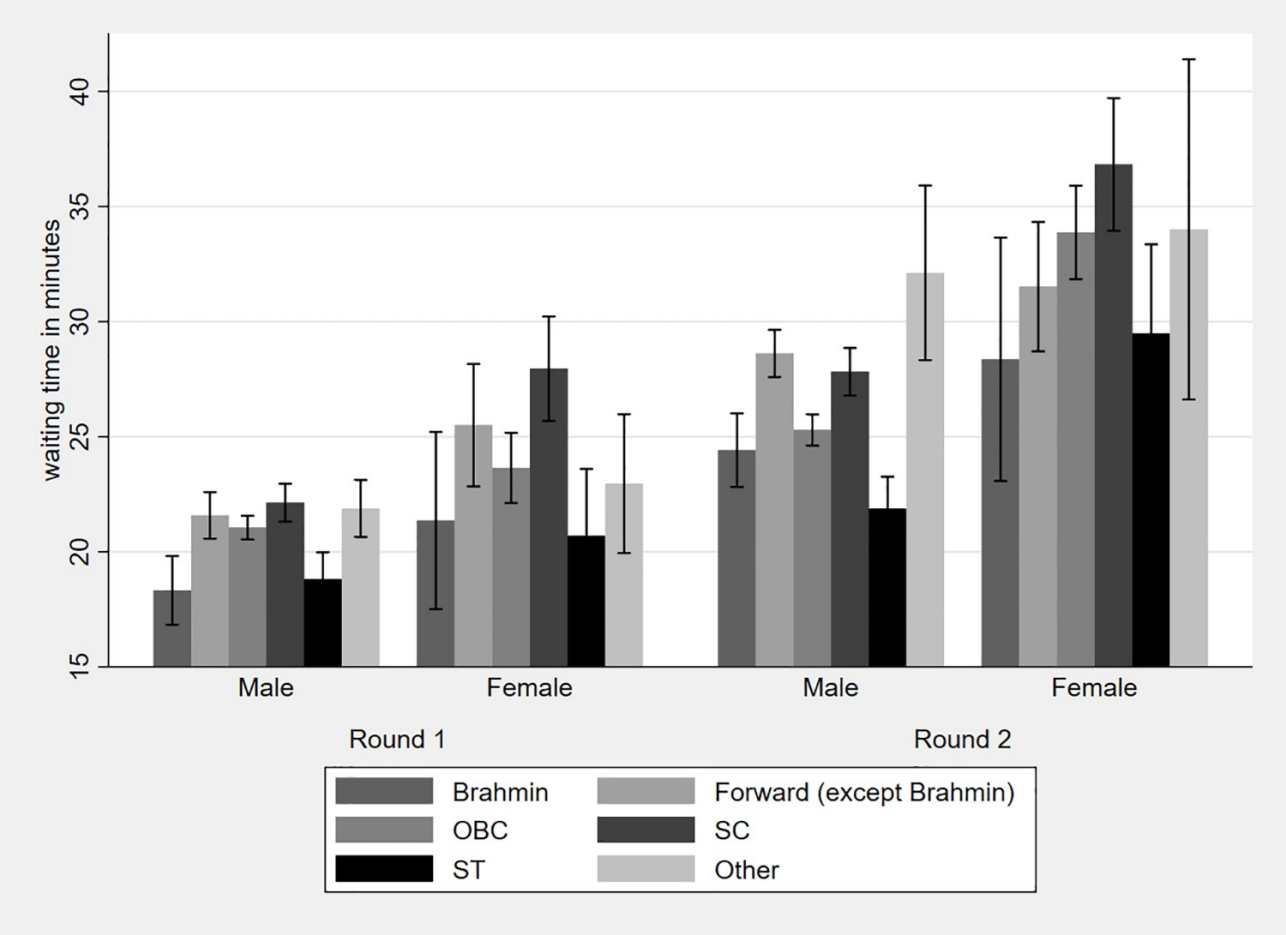
Average waiting time by caste and provider gender.

Fig 3 shows the average waiting time by caste and provider gender for each round along with the 95% CI. Waiting time on average is significantly lower with male providers compared with female providers in both rounds. In the first round, when seen by a male provider we observe significant differences in waiting times between Brahmins and all other castes except STs. In the second round, we again observe significant differences between Brahmins and all other castes, except OBCs and STs. For patients seen by female providers however, there are no significant differences in waiting times between Brahmins and other castes in both rounds of the survey.

**Fig 4:**
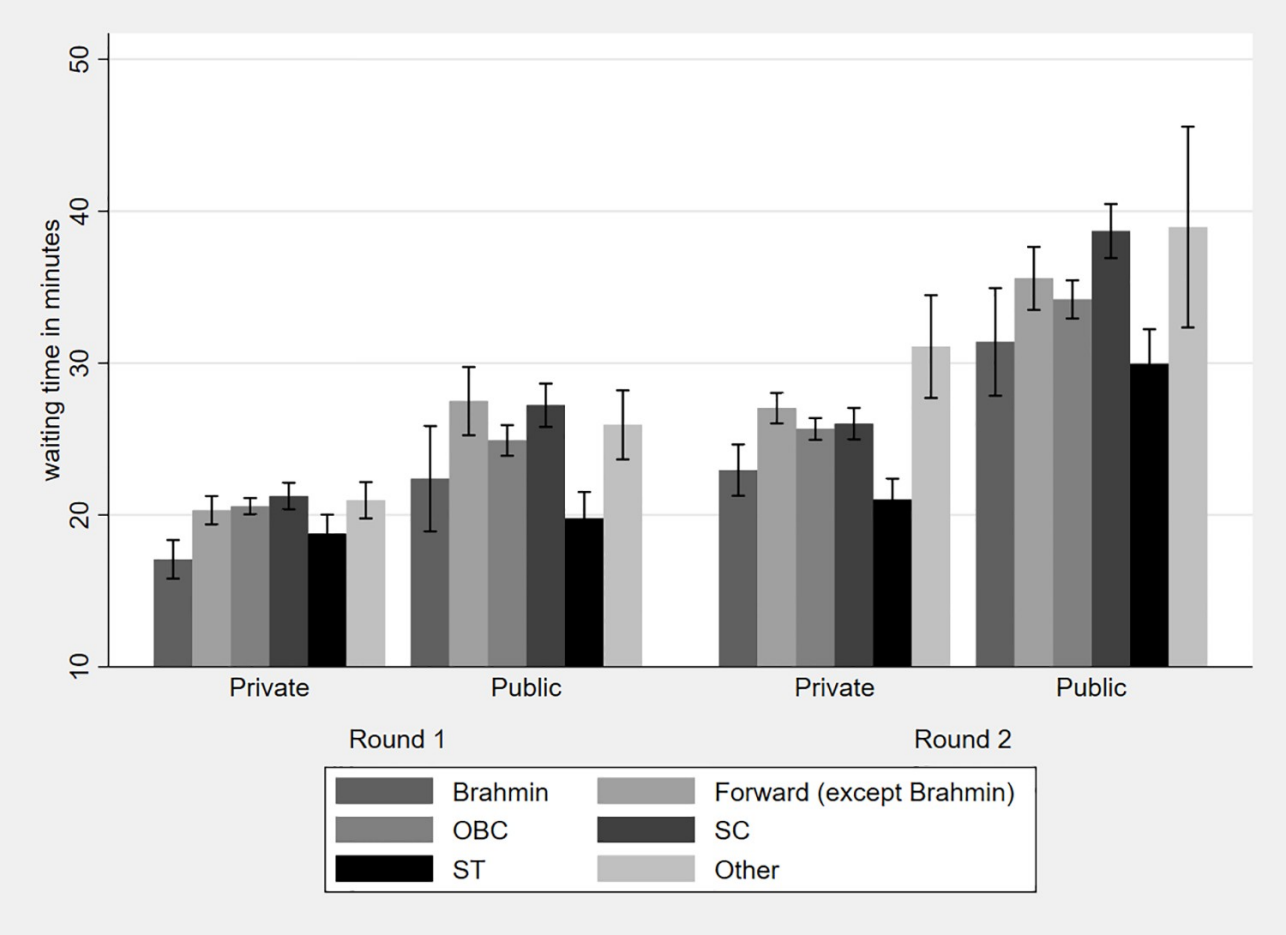
Average waiting time by caste and provider type.

Similarly, Fig 4 shows average waiting time by caste and type of facility visited for each round along with the 95% CI. Waiting times are higher in public than private facilities for each caste in both rounds with waiting times for each facility type increasing from round one to round two. Fig 4 shows that there are significant differences in waiting times between Brahmins and other castes within the private sector in both rounds (with the exception of STs). There are no statistically significant differences though within the public sector in the first round; in the second round there is a significant difference only between Brahmins and SCs.

### Statistical analysis

We are interested in the relationship between caste and waiting time. Our dependent variable of interest is ‘caste’ which is categorical with the reference category being ‘Brahmins’ in all the regressions. We also control for religion, household income, education, place of residence (rural vs. urban) and illness type. All variables are categorical with the exception of income. Observations with missing values in one of the two waves are excluded since we are interested in following the outcome variable for the same households. Moreover, caste is time-invariant hence a regression model using within- variation is not feasible. The actual dependent variable in our analysis is waiting time at a health care facility and therefore restricted to nonnegative integers. However, we employ two different regression models. In a first step, we generate a binary indicator that equals 1 if the respondent reports a waiting time above zero i.e. any waiting time at all; it equals 0 if the respondent reports zero waiting time i.e. did not have to wait at all. We estimate

$${wait\_time\_bin}_{i}=\alpha +\;\beta_{1}\;{caste}_{i}+\;\beta_{2}\;{religion}_{i}+\;\beta_{3}\text{log}\left({income}_{i}\right)+\;\beta_{4}\;{education}_{i}+\;\beta_{5}{\;rural}_{i}+\;\beta_{6}\;{illness}_{i}+\;u_{i}$$

wait_time_bin={1ifwait_time_bin>00ifwait_time_bin≤0

using a logit regression (marginal effects reported in results) with the binary waiting time variable as the dependent variable. Index *i* refers to the responding individual in the household, typically the head of the household. In a second step we use waiting time in minutes, which is not equidispersed as the (conditional) mean is smaller than the (conditional) variance, as the dependent variable. To account for over-dispersion and non-linearity we apply a negative binomial model with robust standard errors. Specifically, we estimate

$${wait\_time}_{i}=\alpha +\;\beta_{1}\;{caste}_{i}+\;\beta_{2}\;{religion}_{i}+\;\beta_{3}\text{log}\left({income}_{i}\right)+\;\beta_{4}\;{education}_{i}+\;\beta_{5}{\;rural}_{i}+\;\beta_{6}\;{illness}_{i}+\;e_{i}$$

We also assess heterogeneity in the results by conducting sub-sample analysis by provider gender and provider type as mentioned earlier. The regressions are estimated for round 1 and 2 separately to assess how the relationship between caste and waiting time evolves between 2005 and 2012. All regressions are clustered at the household level.

## Results

Columns 1 and 2 of Table 2 show results of the relationship between caste and the waiting time dummy for round 1 and round 2 of the IHDS. Overall, in both rounds, relative to Brahmins, all other castes are positively and significantly related (p < 0.0001) to experiencing any waiting time (except STs). In round 1, an individual belonging to the ‘Forward caste’ is 4 percentage points more likely to experience waiting time than a Brahmin (p < 0.0001). Similarly, an individual belonging to another backward caste or scheduled caste is 6 percentage points more likely to experience any waiting time than a Brahmin (p < 0.0001). The likelihood of experiencing any waiting time relative to ‘Brahmins’ is the highest for ‘Other’ castes (7 percentage points) (p < 0.0001). Considering that the baseline probability of having waiting time in round 1 is 90 percent, these marginal effects are sizeable. The same results are observed for round 2, i.e. relative to Brahmins all other castes have a higher likelihood of experiencing any waiting time (p < 0.0001). While the likelihood of experiencing any waiting time for ‘Forward’ caste and ‘Other’ caste is the same in magnitude, it is reduced to half of that of round 1 for ‘Scheduled’ and ‘Other Backward’ castes. Concurrently, the marginal effect for ‘Scheduled Tribe’ while insignificant in round 1 is now significant in round 2 at a 5 percent level. The baseline probability of having any waiting time in round 2 is 94 percent overall, indicating that the association is not only sizeable but the outcome itself has worsened from round 1 to round 2. Columns 3 and 4 of Table 2 show results of the relationship between caste and the waiting time count variable for round 1 and round 2 of the IHDS. As expected, similar results are obtained using a negative binomial model, i.e. relative to ‘Brahmins’ all other castes are positively related to waiting time in minutes. For example, in round 1, ‘Forward’ and ‘Other Backward’ castes have 15 percent higher waiting times relative to ‘Brahmins’ (p < 0.0001), while scheduled castes experience as much as 24 percent higher waiting times (p < 0.0001). Overall, in round 2, the coefficients are lower for all castes with the exception of ‘Other’ castes but still significant (p < 0.0001 for ‘Forward’, ‘Scheduled’ castes and ‘Others’ and p = 0.003 for ‘Other Backward’ castes). For brevity, we do not show the tables with the full set of control variables, however, these are available from the authors upon request.

**Table 2:** Waiting time and caste.

Dependent variable	Waiting time dummy		Waiting time in minutes	
	*Round 1*	*Round 2*	*Round 1*	*Round 2*
	(1)	(2)	(3)	(4)
**Caste (reference is Brahmin)**				
Forward (except Brahmin)	0.0401***	0.0430***	0.155***	0.134***
	(0.010)	(0.00803)	(0.0417)	(0.0348)
Other backward caste	0.064***	0.0318***	0.147***	0.0977***
	(0.00945)	(0.00782)	(0.0379)	(0.0331)
Scheduled caste	0.0569***	0.0398***	0.244***	0.192***
	(0.00993)	(0.00801)	(0.0401)	(0.0348)
Scheduled tribe	-0.00153	0.0208**	0.0373	-0.0590
	(0.0125)	(0.00947)	(0.0473)	(0.0427)
Other	0.0660***	0.0718***	0.140***	0.270***
	(0.0109)	(0.00974)	(0.0436)	(0.0573)
**Control variables**	Yes	Yes	Yes	Yes
Constant	2.242***	1.609***	3.275***	2.960***
	(0.2640)	(0.3251)	(0.0958)	(0.0913)
Observations	27,244	27,224	27,251	27,251

Notes: The models with waiting time dummy as dependent variable are estimated using a logit specification, the ones with waiting time in minutes as dependent variable are estimated using a negative binomial specification. The coefficients of the logit model are to be interpreted as marginal effects. Control variables include level of schooling, net household income, place of residence (rural or urban), type of minor illness, and religion. Robust standard errors in parentheses;

*** p<0.01,

** p<0.05,

* p<0.1

### Heterogeneous effects

We first present heterogeneity in the results by gender of the health care provider and then by type of provider (private or government).

Results in Table 3 show the heterogeneity analysis by gender of provider for both round for the dummy variable and the count waiting time variable. The results are shown for round 1 and round 2 by provider gender and provider type. Columns 1 to 4 show results of the logit model by gender of provider. In both rounds, we find a positive and significant relationship of all castes (relative to ‘Brahmins’) and the waiting time dummy for individuals that see a male health care worker (p < 0.001) with the exception of ‘Scheduled tribe’ (p = 0.291). For female providers, the results are less consistent with insignificant coefficients in round 1 (except ‘Scheduled caste’, p = 0.009) and positively significant coefficients in the second round (p <0.0001, except ST and others where p < 0.002). Columns 5 to 8 in Table 3 show the results of the negative binomial regression with waiting time in minutes as the dependent variable. In both rounds, we find a positive and significant relationship between different castes (relative to ‘Brahmins’) and waiting time only for individuals that see a male health care worker (p = 0.008 for ‘Forward’, p = 0.013 for ‘OBC’, p < 0.0001 for ‘Scheduled caste’). The exception is for patients from ‘scheduled tribes’ that have lower waiting times with regards to ‘Brahmins’, albeit only statistically significant in round 2 (p = 0.007). Results show no significant differences in waiting times across castes for patients that see female providers with the exception of ‘Scheduled’ caste that exhibit a higher waiting time with regards to ‘Brahmin’ (p = 0.036 in both rounds) with the same effect observed for patients of the same caste that see male providers.

**Table 3:** Waiting time and caste: Heterogeneity analysis by provider gender.

Dependent variable	Waiting time dummy				Waiting time in minutes			
	*Round 1*		*Round 2*		*Round 1*		*Round 2*	
	Female	Male	Female	Male	Female	Male	Female	Male
	(1)	(2)	(3)	(4)	(5)	(6)	(7)	(8)
**Caste (reference is Brahmin)**								
Forward (except Brahmin)	0.0285	0.0386***	0.0852***	0.0372***	0.139	0.130***	0.0878	0.144***
	(0.0261)	(0.0120)	(0.0307)	(0.00890)	(0.112)	(0.0490)	(0.107)	(0.0384)
Other forward caste	0.0458	0.0503***	0.0838***	0.0197**	0.0367	0.110**	0.167	0.0204
	(0.0241)	(0.0110)	(0.0300)	(0.00866)	(0.102)	(0.0443)	(0.104)	(0.0366)
Scheduled caste	0.0553**	0.0497***	0.0919***	0.0301***	0.224**	0.214***	0.225**	0.145***
	(0.0248)	(0.0115)	(0.0305)	(0.00890)	(0.106)	(0.0472)	(0.107)	(0.0392)
Scheduled tribe	-0.0525	-0.0158	0.0975***	-0.000853	-0.0887	-0.000480	-0.0520	-0.136***
	(0.0342)	(0.0149)	(0.0330)	(0.0111)	(0.126)	(0.0569)	(0.129)	(0.0508)
Other	0.0472	0.0577***	0.117***	0.0672***	0.00771	0.102**	0.194	0.243***
	(0.0275)	(0.0129)	(0.0321)	(0.0120)	(0.117)	(0.0519)	(0.150)	(0.0748)
**Control variables**	Yes	Yes	Yes	Yes	Yes	Yes	Yes	Yes
Constant	2.496***	1.661***	1.683*	1.409***	3.058***	3.242***	3.467***	2.763***
	(0.869)	(0.296)	(0.869)	(0.371)	(0.283)	(0.115)	(0.254)	(0.113)
Observations	3,295	19,514	3,583	19,217	3,320	19,519	3,602	19,237

Notes: The models with waiting time dummy as dependent variable are estimated using a logit specification, the ones with waiting time in minutes as dependent variable are estimated using a negative binomial specification. The coefficients of the logit model are to be interpreted as marginal effects. Control variables include level of schooling, net household income, place of residence (rural or urban), type of minor illness, and religion. Robust standard errors in parentheses;

*** p<0.01,

** p<0.05,

* p<0.1

Table 4 shows the results for private and government providers separately. In round 1, the probability of waiting (columns 1 and 2) is significantly higher for all castes compared to ‘Brahmins’ in private as well as governmental health care facilities (p < 0.05, except ‘Forward caste’ in a government facility). Similarly, in round 2 all castes except ‘Scheduled tribe’ have a significantly higher probability of waiting then ‘Brahmins’ (p < 0.05), independent of health care provider type. Caste based inequality in length of waiting time (columns 5 to 8) is observed only in the case of private providers, i.e. relative to ‘Brahmins’, all other castes have a statistically significant association with higher waiting time (except ‘scheduled’ caste in round 1) (p < 0.0001 in round 1 and p < 0.05 in round 2). As in the case of provider gender, ‘scheduled tribes’ are negatively related to waiting time but not statistically significant (except in round 2 for private providers). Despite significant inequality in waiting time by caste, the magnitude of the coefficients has reduced by both provider gender and type from round 1 to round 2.

**Table 4:** Waiting time and caste: Heterogeneity analysis by type of provider.

Dependent variable	Waiting time dummy				Waiting time in minutes			
	*Round 1*		*Round 2*		*Round 1*		*Round 2*	
	Govt.	Private	Govt.	Private	Govt.	Private	Govt.	Private
	(1)	(2)	(3)	(4)	(5)	(6)	(7)	(8)
**Caste (reference is Brahmin)**								
Forward (except Brahmin)	0.0371*	0.0407***	0.0336***	0.0476***	0.122	0.176***	0.0991	0.154***
	(0.0199)	(0.0119)	(0.0119)	(0.0103)	(0.0884)	(0.0441)	(0.0636)	(0.0414)
Other forward caste	0.0967***	0.0499***	0.0317***	0.0316***	0.0398	0.200***	0.0810	0.0974**
	(0.0178)	(0.0111)	(0.0115)	(0.0100)	(0.0794)	(0.0402)	(0.0584)	(0.0401)
Scheduled caste	0.0915***	0.0414***	0.0333***	0.0398***	0.178**	0.264***	0.209***	0.149***
	(0.0182)	(0.0118)	(0.0117)	(0.0104)	(0.0812)	(0.0439)	(0.0602)	(0.0426)
Scheduled tribe	0.0535**	-0.0349**	0.0253*	0.0116	-0.0973	0.0883*	-0.0712	-0.111**
	(0.0211)	(0.0158)	(0.0132)	(0.0127)	(0.0930)	(0.0530)	(0.0716)	(0.0517)
Other	0.0848***	0.0564***	0.0480***	0.0789***	0.0351	0.181***	0.210**	0.245***
	(0.0202)	(0.0128)	(0.0126)	(0.0140)	(0.0885)	(0.0477)	(0.101)	(0.0644)
**Control variables**	Yes	Yes	Yes	Yes	Yes	Yes	Yes	Yes
Constant	3.446***	1.779***	2.933***	1.271***	3.969***	2.932***	3.510***	2.632***
	(0.526)	(0.310)	(0.880)	(0.356)	(0.176)	(0.111)	(0.153)	(0.110)
Observations	8,035	19,191	8,412	18,688	8,039	19,194	8,524	18,709

Notes: The models with waiting time dummy as dependent variable are estimated using a logit specification, the ones with waiting time in minutes as dependent variable are estimated using a negative binomial specification. The coefficients of the logit model are to be interpreted as marginal effects. Control variables include level of schooling, net household income, place of residence (rural or urban), type of minor illness, and religion. Robust standard errors in parentheses;

*** p<0.01,

** p<0.05,

* p<0.1

### Sensitivity analysis

We observe a change in the caste from round 1 to round 2 of a small fraction of individuals in our data. Technically, one can use within-individual changes in caste to more clearly segregate the effect of interest. However, we believe this is not an ideal strategy since we are unaware of the exact reason for this caste switch i.e. input error in round 1 or round 2, a meaningful change in caste, or simply selection by individuals. For these reasons we conduct robustness checks by excluding such cases without speculating on the reason for the change. The results are similar to those in the main analysis (except for ‘Scheduled Tribe’). Further, since caste is primarily a feature of the Hindu religion, as an additional robustness test we restrict the sample only to Hindus and find the same results. These results are presented in Tables C and D in S1 Appendix.

## Discussion

Responsiveness of health care systems, i.e. meeting legitimate expectations of individuals, is an important goal proposed by the WHO. Not only is the level of responsiveness crucial but also its distribution is of utmost importance since a more responsive system leads to better health by enhancing utilization, especially if patients anticipate being treated well and in a timely manner [43]. In this paper, we assess the relationship between social class i.e. caste of an individual and waiting time at health facilities—a client orientation dimension of responsiveness. We find that lower social caste is associated with higher waiting time in minutes. This relationship is consistently significant for individuals that visited a male provider but not so much if they visited a female provider. Further, caste is positively related to the length of waiting time only if seen in a private facility; for individuals visiting a government facility the relationship between waiting time and caste is not significant. The results are robust to the inclusion of several covariates, different types of regression models and sensitivity tests.

Equity in all frontiers of life is one of the primary objectives of any social system; yet, disparities related to health care based on castes or ethnic groups still exist. In particular, the literature on inequalities in responsiveness of health systems is scarce with respect to issues in developing countries. While socio-economic indicators such as income and education are considered crucial in determining the level and distribution of responsiveness of health systems in general, different countries (especially developing countries) may have different factors that might determine responsiveness. It is important therefore to take into account country specific factors e.g. the cultural context that may well be responsible for inequality in responsiveness indicators. The results show that the factors that may determine waiting time may be different in different contexts and accounting for such differences in cross country studies is important if policies to improve health are to be well informed. Even within a country (where people may belong to the same ethnicity) inequality may arise due to other culture specific discriminatory practices. This is not only true in the Indian context but also applicable to other developed and developing countries across the world where there is segmentation within a society.

### Limitations

While we believe that our paper makes an important contribution towards highlighting the importance of cultural context in relation to health, there are however several caveats of our analysis. While waiting time is an objective indicator in general, in the survey it is self-reported [44]. If lower castes are less likely to report higher waiting times simply due to different expectations compared to higher castes, then we may be underestimating the true effect. Another caveat of the analysis is that we assess the relation between caste and waiting time conditional on having utilized a health care facility. However, it is important to acknowledge that those that do not have access to health care or do not make use of it even when in need are most likely to be discriminated on the basis of caste. Also, we assume that the healthcare worker can observe the caste of the individual, which in this setting is a reasonable assumption. Finally, while we cannot directly control for severity of illness due to lack of data, we do account for the type of illness for which the health facility was visited.

## Supporting information

S1 Appendix Table A: t-test for waiting time Round 1 versus Round 2. Table B: Difference in waiting time by caste categories. Table C: Waiting time and caste—sample restricted to caste non-switchers. Table D: Waiting time and caste—sample restricted to Hindus.
(DOCX)
