# Supplementary material for: Waiting time at health facilities and social class: Evidence from the Indian caste system
Source: PLoS One. 2018 Oct 15;13(10):e0205641. doi: 10.1371/journal.pone.0205641 (PMC6188850; doi:10.1371/journal.pone.0205641)
Supplement: S1 Appendix — Table A: t-test for waiting time Round 1 versus Round 2. Table B: Difference in waiting time by caste categories. Table C: Waiting time and caste—sample restricted to caste non-switchers. Table D: Waiting time and caste—sample restricted to Hindus. (DOCX) [file pone.0205641.s001.docx]

**S1 Appendix**

**Table A: t-test for waiting time Round 1 versus Round 2**

|  | **Obs.** | **Mean** | **Std. Err** |
| --- | --- | --- | --- |
| **Group** |  |  |  |
| Round 1 | 27,251 | 21.82254 | 0.1663368 |
| Round 2 | 27,251 | 28.62053 | 0.2118147 |
| Difference |  | -6.797989 | 0 .2693203 |
|  |  |  |  |
| **t-statistic** | -25.2413 | | |
|  |  |  |  |
| **Ho: Difference = 0** | Ha: diff < 0: Pr(T < t) = 0.0000 | | |
|  | Ha: diff != 0: Pr(\|T\| > \|t\|) = 0.0000 | | |
|  | Ha: diff > 0: Pr(T > t) = 1.0000 | | |

**Table B: Difference in waiting time by caste categories**

| **Comparison of waiting time by caste (Bonferroni)** | | | | | |
| --- | --- | --- | --- | --- | --- |
| *Row Mean minus Column Mean* |  |  |  |  |  |
|  | **Brahmin** | **Forward** | **OBC** | **SC** | **ST** |
| **Forward** | 4.43341 |  |  |  |  |
|  | (0.0000) |  |  |  |  |
| **OBC** | 3.13867 | -1.29473 |  |  |  |
|  | (0.0000) | (0.008) |  |  |  |
| **SC** | 4.87606 | 0.442652 | 1.73739 |  |  |
|  | (0.0000) | (1.000) | (0.000) |  |  |
| **ST** | 0.10295 | -4.33046 | -3.03572 | -4.77311 |  |
|  | (1.0000) | (0.000) | (0.000) | (0.000) |  |
| **Other** | 2.43252 | -2.00088 | -0.70615 | -2.44354 | 2.32957 |
|  | (0.056) | (0.046) | (1.000) | (0.004) | (0.039) |

Bonferroni-adjusted significance in brackets

**Table C: Waiting time and caste - sample restricted to caste non-switchers**

| Dependent variable | **Waiting time dummy** | | **Waiting time in minutes** | |
| --- | --- | --- | --- | --- |
|  | *Round 1* | *Round 2* | *Round 1* | *Round 2* |
|  | (1) | (2) | (3) | (4) |
| **Caste (reference is Brahmin)** |  |  |  |  |
| Forward (except Brahmin) | 0.0492*** | 0.0416*** | 0.158*** | 0.131*** |
|  | (0.0116) | (0.00905) | (0.0481) | (0.0399) |
| Other backward caste | 0.0693*** | 0.0310*** | 0.156*** | 0.0854** |
|  | (0.0109) | (0.00867) | (0.0450) | (0.0369) |
| Scheduled caste | 0.0676*** | 0.0383*** | 0.265*** | 0.215*** |
|  | (0.0114) | (0.00889) | (0.0472) | (0.0389) |
| Scheduled tribe | 0.00391 | 0.0109 | 0.0335 | -0.108** |
|  | (0.0143) | (0.0108) | (0.0559) | (0.0491) |
| Other | 0.100*** | 0.0656*** | 0.325*** | 0.240*** |
|  | (0.0198) | (0.0133) | (0.0819) | (0.0872) |
|  |  |  |  |  |
| **Control variables** | Yes | Yes | Yes | Yes |
|  |  |  |  |  |
| Constant | 2.161*** | 1.5242*** | 3.182*** | 2.929*** |
|  | (0.291) | 0.3598 | (0.109) | (0.104) |
| Observations | 21,685 | 21,671 | 21,692 | 21,692 |

Notes: The models with waiting time dummy as dependent variable are estimated using a logit specification, the ones with waiting time in minutes as dependent variable are estimated using a negative binomial specification. The coefficients of the logit model are to be interpreted as marginal effects. Control variables include level of schooling, net household income, place of residence (rural or urban), type of minor illness, and religion. Robust standard errors in parentheses; *** p<0.01, ** p<0.05, * p<0.1

**Table D: Waiting time and caste - sample restricted to Hindus**

|  | Waiting time dummy | | Waiting time in minutes | |
| --- | --- | --- | --- | --- |
| Dependent variable | *Round 1* | *Round 2* | *Round 1* | *Round 2* |
| **Caste (reference is Brahmin)** |  |  |  |  |
|  |  |  |  |  |
| Forward (except Brahmin) | 0.0346*** | 0.0460*** | 0.113** | 0.103*** |
|  | (0.0115) | (0.00859) | (0.0438) | (0.0359) |
| Other backward caste | 0.0751*** | 0.0366*** | 0.159*** | 0.102*** |
|  | (0.0103) | (0.00827) | (0.0381) | (0.0336) |
| Scheduled caste | 0.0665*** | 0.0437*** | 0.256*** | 0.209*** |
|  | (0.0109) | (0.00850) | (0.0405) | (0.0353) |
| Scheduled tribe | 0.000933 | 0.0212** | 0.0571 | -0.0468 |
|  | (0.0137) | (0.0101) | (0.0490) | (0.0447) |
| Other | 0.0751*** | 0.0890*** | 0.116** | 0.331*** |
|  | (0.0124) | (0.00902) | (0.0463) | (0.0665) |
|  |  |  |  |  |
| **Control variables** | Yes | Yes | Yes | Yes |
|  |  |  |  |  |
| Constant | 2.1556*** | 1.6190*** | 3.319*** | 2.983*** |
|  | (0.2806) | (0.3572) | (0.105) | (0.101) |
| Observations | 22,066 | 22,215 | 22,066 | 22,215 |

Notes: The models with waiting time dummy as dependent variable are estimated using a logit specification, the ones with waiting time in minutes as dependent variable are estimated using a negative binomial specification. The coefficients of the logit model are to be interpreted as marginal effects. Control variables include level of schooling, net household income, place of residence (rural or urban), type of minor illness, and religion. Robust standard errors in parentheses; *** p<0.01, ** p<0.05, * p<0.1
